# Non-contiguous finished genome sequence and description of *Fenollaria massiliensis* gen. nov., sp. nov., a new genus of anaerobic bacterium

**DOI:** 10.4056/sigs.3957647

**Published:** 2014-02-15

**Authors:** Isabelle Pagnier, Olivier Croce, Catherine Robert, Didier Raoult, Bernard La Scola

**Affiliations:** Unité de Recherche sur les Maladies Infectieuses et Tropicales Emergentes, Faculté de médecine, Aix-Marseille Université, France

**Keywords:** *Fenollaria massiliensis*, genome

## Abstract

*Fenollaria massiliensis* strain 9401234^T^, is the type strain of *Fenollaria massiliensis* gen. nov., sp. nov., a new species within a new genus *Fenollaria*. This strain, whose genome is described here, was isolated from an osteoarticular sample. *F. massiliensis* strain 9401234^T^ is an obligate anaerobic Gram-negative bacillus. Here we describe the features of this organism, together with the complete genome sequence and annotation. The 1.71 Mbp long genome exhibits a G+C content of 34.46% and contains 1,667 protein-coding and 30 RNA genes, including 3 rRNA genes.

## Introduction

*Fenollaria massiliensis* strain 9401234^T^ (= CSUR P127 = DSM 26367), is the type strain of *Fenollaria massiliensis* sp. nov., and the first member of the new genus *Fenollaria* gen. nov. This bacterium is a Gram-negative, anaerobic, non spore-forming, indole positive bacillus that was isolated from an osteoarticular sample, during a study prospecting anaerobic isolates from deep samples [1].

Traditionally, definition of a new bacterial species or genus has relied on the application of the “gold standard” methods of DNA-DNA hybridization and G+C content determination [2]. However, those methods are expensive, and poorly reproducible. The development of PCR and sequencing methods led to new ways of classifying bacterial species, using, in particular, 16S rRNA sequences with cutoff [3], together with phenotypic characteristics. Recently, a number of new bacterial genera and species have been described using high throughput genome sequencing and mass spectrometric analyses, which allows access to a wealth of genetic and proteomic information [4,5]. We propose a new bacterial genus and species using a whole genome sequence and a MALDI-TOF spectrum, and the main characteristics of the organism, as we have previously done [6-12].

Here we present a summary classification and a set of features for *F. massiliensis* gen. nov., sp. nov. strain 9401234^T^ (= CSUR P127= DSM 26367) together with the description of the complete genomic sequencing and annotation. These characteristics support the circumscription of a novel genus, *Fenollaria* gen. nov., within the *Clostridiales* Family XI Incertae sedis, with *Fenollaria massiliensis* gen. nov., sp. nov, as the type species.

*Clostridiales* Family XI Incertae sedis was created in 2009 [13], and currently comprises 11 genera, including *Anaerococcus*, *Peptoniphilus* and *Tissierella*. It is a heterogeneous group that includes anaerobic and morphologically variable bacteria. This group is defined mainly on the basis of phylogenetic analyses of 16S rRNA sequences and its members have no precise taxonomic or phylogenetic affiliation. Based on the 16S rRNA comparison, the species most closely related to *Fenollaria massiliensis* is *Sporobacterium olearium* [14], which is the sole representative of the genus *Sporobacterium*. *S. olearium* is a Gram-positive rod with terminal spores. The most closely related validly named species is *Tissierella creatinini*, which belongs to the genus *Tissierella* sp [15]. It was first described in 1986 and is represented by three species, among which the type species is *T. praecuta,* a strictly anaerobic Gram-negative, non spore-forming bacterium.

## Classification and features

**Figure 1:**
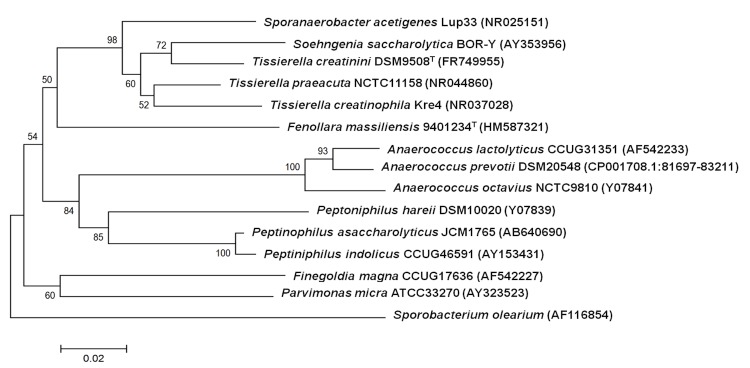
Phylogenetic tree highlighting the position of *Fenollaria massiliensis* strain 9401234^T^ relative to other type strains within the *Clostridiales* Family XI Incertae sedis. GenBank accession numbers are indicated in parentheses. Sequences were aligned using CLUSTALW, and phylogenetic inferences obtained using the maximum-likelihood method within the MEGA 4 software [27]. Numbers at the nodes are bootstrap values obtained by repeating the analysis 500 times to generate a majority consensus tree. The scale bar represents a 2% nucleotide sequence divergence.

An osteoarticular sample was collected from a patient as part of a study analyzing emerging anaerobic infectious agents by MALDI-TOF and 16S rRNA gene sequencing. The specimen was sampled in Marseille and preserved at -80°C after collection. Strain 9401234^T^ (Table 1) was isolated in February 2009, by anaerobic cultivation on 5% sheep blood-enriched Columbia agar (BioMerieux, Marcy l’Etoile, France). Based on the 16S rRNA sequencing, this strain exhibited 87% sequence similarity with *Tissierella creatinini* [26]. In the inferred phylogenetic tree, it forms a distinct lineage within the *Clostridiales* Family XI Incertae sedis (Figure 1). Those similarity values are lower than the recommended threshold to delineate a new genus without carrying out DNA-DNA hybridization [3].

**Table 1:** Classification and general features of *Fenollaria massiliensis* strain 9401234^T^ according to the MIGS recommendations [16]

**MIGS ID**	**Property**	**Term**	**Evidence code^a^**
		Domain *Bacteria*	TAS [17]
		Phylum *Firmicutes*	TAS [18-20]
		Class *Clostridia*	TAS [21,22]
	Current classification	Order *Clostridiales*	TAS [23,24]
		Family XI Incertae sedis	TAS [13]
		Genus *Fenollaria*	IDA
		Species *Fenollaria massiliensis*	IDA
		Type strain 9401234^T^	IDA
	Gram stain	Negative	IDA
	Cell shape	Rod-shaped	IDA
	Motility	Non motile	IDA
	Sporulation	Non spore-forming	IDA
	Temperature range	Mesophile	IDA
	Optimum temperature	37°C	IDA
MIGS-6.3	Salinity	Weak growth on BHI agar + 1% NaCl	IDA
MIGS-22	Oxygen requirement	Anaerobic	IDA
	Carbon source	Unknown	NAS
	Energy source	Unknown	NAS
MIGS-6	Habitat	Human	IDA
MIGS-15	Biotic relationship	Free living	IDA
MIGS-14	Pathogenicity	Unknown	NAS
	Biosafety level	2	
	Isolation	Osteoarticular sample	
MIGS-4	Geographic location	France	IDA
MIGS-5	Sample collection time	February 2009	IDA
MIGS-4.1	Latitude	43.296482	IDA
MIGS-4.1	Longitude	5.36978	IDA
MIGS-4.3	Depth	Surface	IDA
MIGS-4.4	Altitude	0 above see level	IDA

**^a^**Evidence codes - IDA: Inferred from Direct Assay; TAS: Traceable Author Statement (i.e., a direct report exists in the literature); NAS: Non-traceable Author Statement (i.e., not directly observed for the living, isolated sample, but based on a generally accepted property for the species, or anecdotal evidence). These evidence codes are from the Gene Ontology project [25]. If the evidence is IDA, then the property was directly observed for a live isolate by one of the authors or an expert mentioned in the acknowledgements.

Growth at different temperatures was tested; no growth occurred at 23°C, 25°C, 28°C and 50°C, but did occur between 32° and 37°C. Optimal growth was observed at 37°C.

**Figure 2:**
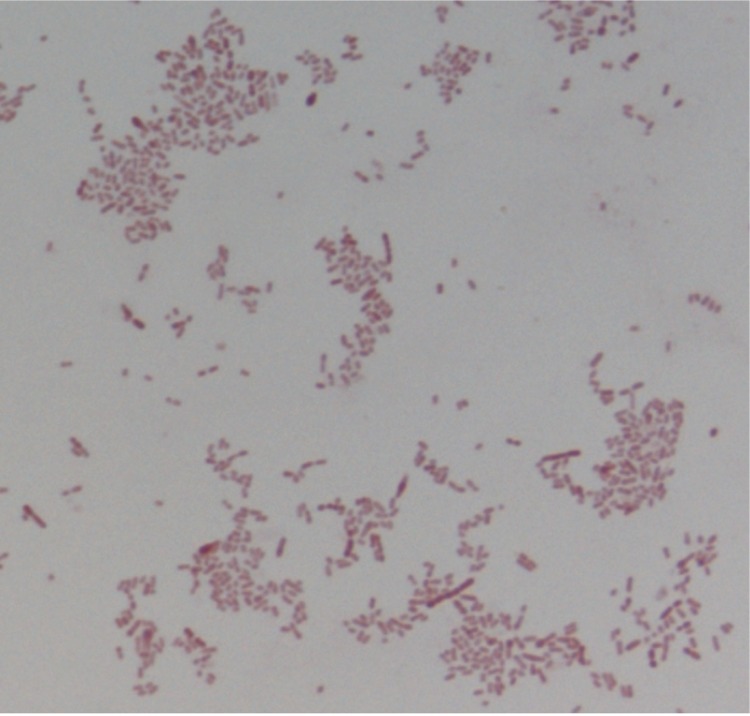
Gram stain of *F. massiliensis* strain 9401234^T^

**Figure 3:**
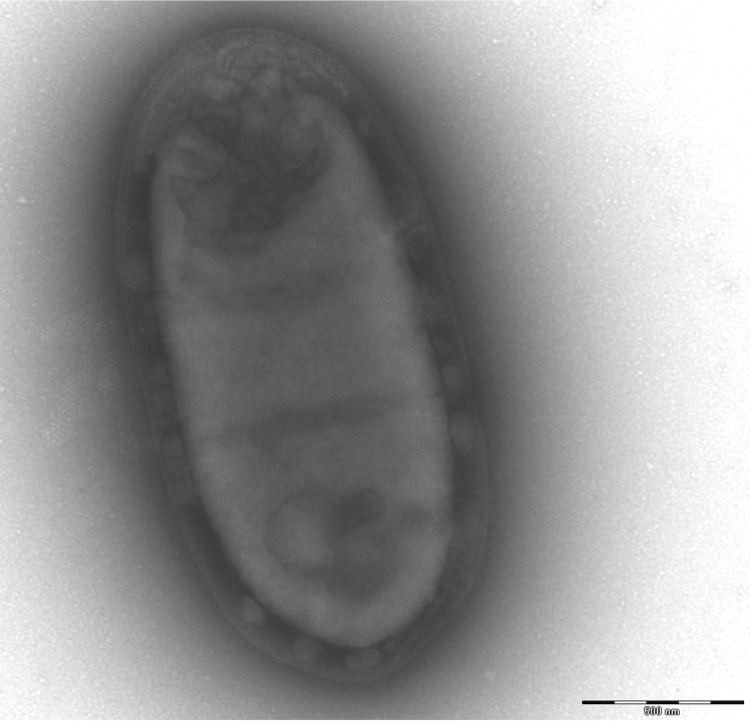
Transmission electron micrograph of *F. massiliensis* strain 9401234^T^, taken using a Morgani 268D (Philips) at an operating voltage of 60kV. The scale bar represents 500 nm.

Colonies are punctiform, grey, smooth, and round when grown on blood-enriched Columbia agar (Biomerieux), under anaerobic conditions using GENbag anaer (BioMérieux). Growth was achieved anaerobically, on blood-enriched Columbia agar and in TS broth medium after 72h. They also were grown under anaerobic conditions on BHI agar supplemented with 1% NaCl. Growth did not occur under microaerophilic conditions and in the presence of air, with 5% CO_2._ . Gram staining showed rod-shaped non spore-forming Gram-negative bacilli (Figure 2). Cells were non-motile. Cells grown in TS broth medium have a mean length of 1.555 µm (min = 1.167µm; max = 2.948µm), and a mean width of 0.772 µm (min = 0.602 µm; max = 1.014 µm), as determined using electron microscopic observation after negative staining (Figure 3).

Strain 9401234^T^ exhibited neither catalase nor oxidase activities. Using the API 20A system, a positive reaction was observed only for indole, and weakly for gelatinase. Using the API Zym system, a positive reaction was observed for leucine arylamidase and valine arylamidase regarding the proteases, and for Naphtol phosphatase. API RapidID 32A confirmed the positivity for indole and leucine arylamidase, and was also positive for arginine arylamidase, and weakly positive for pyrrolidonyl arylamidase, tyrosine arylamidase, glycine arylamidase, histidine arylamidase and serine arylamidase. Regarding antibiotic susceptibility, *F. massiliensis* was susceptible to penicillin G, amoxicillin, cefotetan, imipenem, metronidazole, and vancomycin. When compared to the species *Tissierela creatinini*, *Sporobacterium olearium*, and *Anaerococcus prevotii,* within the *Clostridiales* Family XI *Incertae sedis*, *F. massiliensis* exhibits the phenotypic characteristics details in Table 2.

**Table 2:** Differential characteristics of *Fenollaria massiliensis* gen. nov., sp. nov., strain 9401234^T^, *Tissierela creatinini* strain DSM 9508^T^ [26], *Sporobacterium olearium* strain SR1^T^ [14] and *Anaerococcus prevotii* strain [28].

**Properties**	*F. massilliensis*	*T. creatinini*	*S. olearium*	*A. prevotii*
Cell diameter (µm)	0.6-1/1.2-2.9	1/3.5	0.4-0.8/5-10	0.6/0.9
				
Gram stain	Negative	Positive	Positive	Positive
Salt requirement	-	+	0-30g NaCl/l	na
Motility	-	+	+	-
Endospore formation	-	-	+	-
Optimal growth temperature	37°C	37°C	37-40°C	37°C
Phosphatase	Naphtholphosphatase	Na	na	-
Indole	+	-	na	-
Gelatinase	+	-	na	na
Urease	-	-	na	+
**Utilization of**				
D-Glucose	-	-	-	+
D-mannose	-	-	na	+
Habitat	Human	Environment	Environment	Human

**Figure 4:**
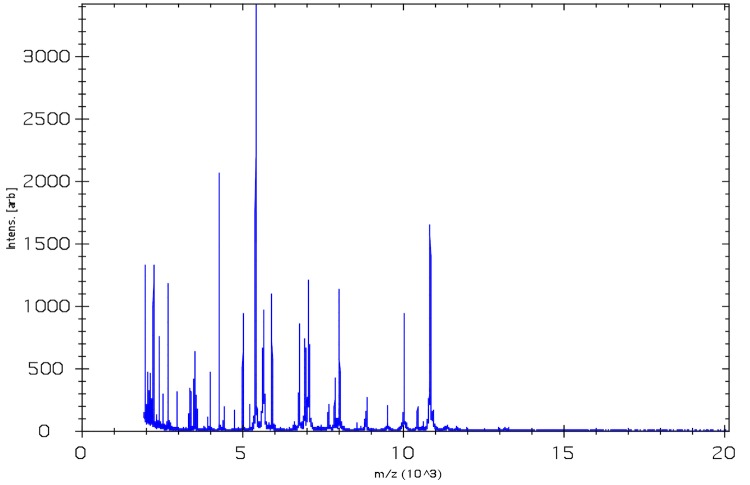
Reference mass spectrum from *F. massiliensis* strain 9401234^T^. Spectra from 10 individual colonies were compared and a reference spectrum was generated.

**Figure 5:**
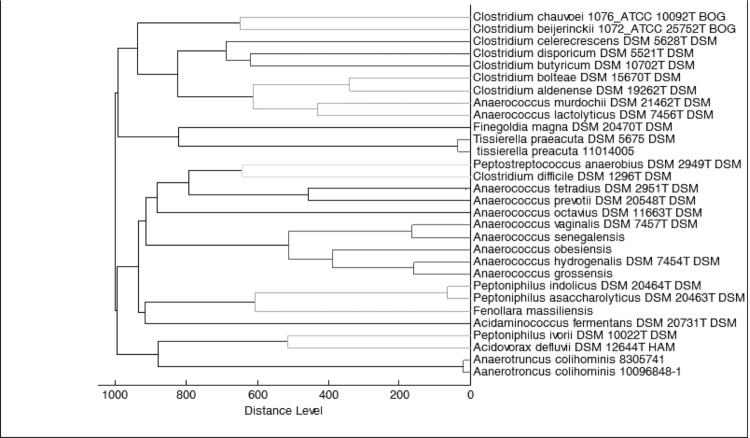
Dendrogram based on the comparison of the *F. massiliensis* strain 9401234^T^ MALDI-TOF reference spectrum, and 29 other species of the order of *Clostridiales*.

Matrix-assisted laser-desorption/ionization time-of-flight (MALDI-TOF) MS protein analysis was carried out as previously described [29]. A pipette tip was used to pick one isolated bacterial colony from a culture agar plate, and to spread it as a thin film on a MTP 384 MALDI-TOF target plate (Bruker Daltonik GmbH, Germany). Ten distinct deposits were done for strain JC122^T^ from ten isolated colonies. Each smear was overlaid with 2µL of matrix solution (saturated solution of alpha-cyano-4-hydroxycinnamic acid) in 50% acetonitrile, 2.5% tri-fluoracetic acid, and allowed to dry for five minutes. Measurements were performed with a Microflex spectrometer (Bruker). Spectra were recorded in the positive linear mode for the mass range of 2,000 to 20,000 Da (parameter settings: ion source 1 (ISI), 20kV; IS2, 18.5 kV; lens, 7 kV). A spectrum was obtained after 675 shots at a variable laser power. The time of acquisition was between 30 seconds and 1 minute per spot. The ten 9401234^T^ spectra were imported into the MALDI Biotyper software (version 2.0, Bruker) and analyzed by standard pattern matching (with default parameter settings) against the main spectra of 5,697 bacteria in the Biotyper database. The method of identification includes the m/z from 3,000 to 15,000 Da. For every spectrum, 100 peaks at most were taken into account and compared with the spectra in database. The output score enabled the identification of the tested species: a score ≥ 2 with a validated species enabled the identification at the species level; a score ≥ 1.7 but < 2 enabled the identification at the genus level; a score < 1.7 was not significant. For strain 9401234^T^, the obtained score was 1.04, which is not significant, suggesting that our isolate was not a member of a known genus. We added the spectrum from strain 9401234^T^ (Figure 4) to our database. A dendrogram was constructed with the MALDI Biotyper software, comparing the reference spectrum of strain 9401234^T^ with reference spectra of 29 bacterial species, all belonging to the order of *Clostridiales* (Figure 5). In this dendrogram, strain 9401234^T^ appears in a separate clade between the genus *Peptoniphilus* and *Acidaminococcus* (Figure 5).

## Genome sequencing and annotation

### Genome project history

The organism was selected for sequencing on the basis of its phylogenetic position, 16S rRNA similarity to other members of the *Clostridiales* Family XI *Incertae sedis*, and its isolation from an osteoarticular clinical sample. It is the first genome of the new genus *Fenollaria* (Genbank accession numbers are CALI02000001-CALI02000010) and consists of 11 contigs. Table 3 shows the project information and its association with MIGS version 2.0 compliance.

**Table 3:** Project information

**MIGS ID**	**Property**	**Term**
MIGS-31	Finishing quality	Non-contiguous finished
MIGS-28	Libraries used	One 454 PE 3-kb library
MIGS-29	Sequencing platforms	454 GS FLX Titanium
MIGS-31.2	Sequencing coverage	19.7
MIGS-30	Assemblers	Newbler 2.6
MIGS-32	Gene calling method	Prodigal 2.5
	Genbank ID	CALI02000001-CALI02000010
	Genbank Date of Release	October 9, 2013
MIGS-13	Source material identifier	DSM 26367
	Project relevance	Study of anaerobic isolates from clinical samples

### Growth conditions and DNA isolation

*F. massiliensis* sp. nov., gen. nov. strain 9401234^T^, CSUR P127 = DSM 26367, was grown on blood agar medium at 37°C under anaerobic conditions. Ten petri dishes were spread and resuspended in 5×100µl of G2 buffer (EZ1 DNA Tissue kit, Qiagen). A first mechanical lysis was performed by glass powder on the Fastprep-24 device (Sample Preparation system) from MP Biomedicals, USA) using 2×20 seconds cycles. DNA was then treated with 2.5 µg/µL lysozyme (30 minutes at 37°C) and extracted through the BioRobot EZ 1 Advanced XL (Qiagen). The DNA was then concentrated and purified on a Qiamp kit (Qiagen). The yield and the concentration were measured by the Quant-it Picogreen kit (Invitrogen) on the Genios_Tecan fluorometer at 135 ng/µl.

### Genome sequencing and assembly

This project was loaded twice on a one-quarter region for the paired end application on PTP Picotiter plates. DNA (5µg) was mechanically fragmented on a Hydroshear device (Digilab, Holliston, MA, USA) with an enrichment size at 3-4kb. The DNA fragmentation was visualized through an Agilent 2100 BioAnalyzer on a DNA LabChip 7500 with an optimal size of 4.2 kb. The library was constructed according to the 454_Titanium paired end protocol and manufacturer recommendations. Circularization and nebulization were performed and generated a pattern with an maximum at 686 bp. After PCR amplification through 15 cycles followed by double size selection, the single stranded paired end library was then quantified on the Agilent 2100 BioAnalyzer with a RNA 6000 Pico chip at 1,820 pg/µL. The library concentration equivalence was calculated as 4.87E+09 molecules/µL. The library was stored at -20°C.

The paired end library was clonal amplified with 1cpb in 3 emPCR reactions with the GS Titanium SV emPCR Kit (Lib-L) v2. The yield of the emPCR was 10.5% according to the quality expected by the range of 5 to 20% from the Roche procedure. 790,000 beads were loaded on the GS Titanium PicoTiterPlates PTP Kit 70×75 sequenced with the GS Titanium Sequencing Kit XLR70. The run was performed overnight and then analyzed on the cluster through the gsRunBrowser and gsAssembler_Roche.

The 454 sequencing generated 119,791 reads (38,34 Mb) and was assembled into contigs and scaffolds using Newbler version 2.6 (Roche) and SSPACE software v1.0 [30] combined with GapFiller V1.10 [31]. A sequence consisting of 6,257,638 reads generated from a SOLiD version 4 with a library constructed through an insert size of 150 bp and a 85 bp (50bp and 35bp) in a paired-end sequencing (Life Technologies) helped to improve the genome assembly using CLC Genomics Workbench v4.7.2 (CLC bio, Aarhus, Denmark). Finally, the available genome consists of 8 scaffolds and 11 contigs.

### Genome annotation

Non-coding genes and miscellaneous features were predicted using RNAmmer [32], ARAGORN [33], Rfam [34] and signalP [35]. Open Reading Frames (ORFs) were predicted using Prodigal [36] with default parameters but the predicted ORFs were excluded if they were spanning a sequencing GAP region. The functional annotation was achieved using BLASTP [37] against the GenBank database [23] and the Clusters of Orthologous Groups (COG) database.

## Genome properties

**Figure 6:**
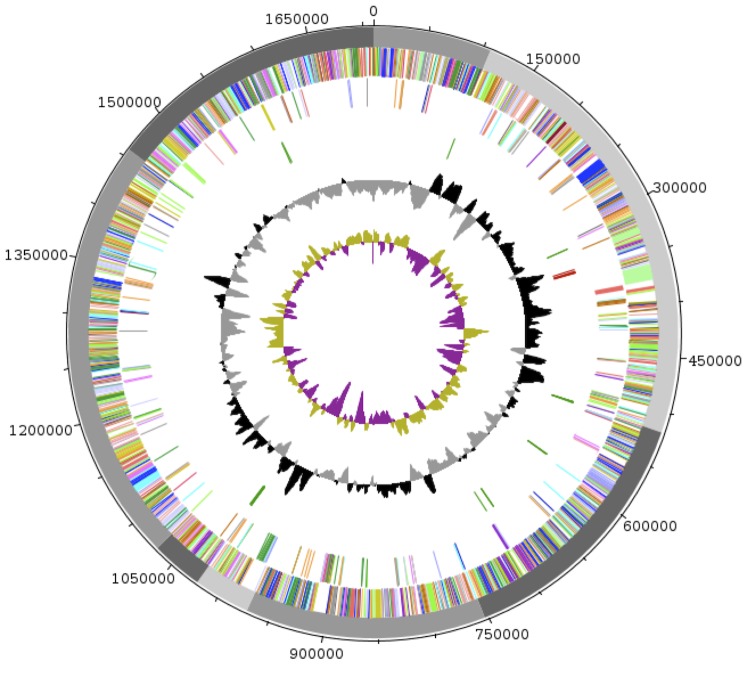
Graphical circular map of the genome. From outside to the center: scaffolds are in grey (unordered), genes on forward strand (colored by COG categories), genes on reverse strand (colored by COG categories), RNA genes (tRNAs green, rRNAs red, other RNAs black), GC content (black/grey), and GC skew (purple/olive).

The genome of *Fenollaria massiliensis* sp. nov. strain 9401234^T^ is estimated at 1.71 Mb long with a G+C content of 36.47% (Figure 6 and Table 4). A total of 1,667 protein-coding and 30 RNA genes, including 3 rRNA genes, 26 tRNA and 1 tmRNA were found. The majority of the protein-coding genes (70.8%) were assigned a putative function while the remaining ones were annotated as hypothetical proteins. The properties and the statistics of the genome are summarized in Table 4 and Table 5.

**Table 4:** Genome statistics

Attribute	Value	% of Total*
Genome size (bp)	1,709,674	100
DNA coding region (bp)	1,554,900	90.9
DNA G+C content (bp)	589,201	34.46
Total genes	1697	100
rRNA genes	3	0.18
tRNA genes	26	1.53
tmRNA	1	0.06
Protein-coding genes	1667	98.23
Genes with function prediction	1180	70.8
Genes assigned to COGs	1744	98.44

* The total is based on either the size of the genome in base pairs or the total number of protein coding genes in the annotated genome

**Table 5:** Number of genes associated with the 25 general COG functional categories

**Code**	**Value**	**%age**^a^	**Description**
J	172	9.86	Translation
A	4	0.23	RNA processing and modification
K	109	6.25	Transcription
L	132	7.57	Replication, recombination and repair
B	4	0.23	Chromatin structure and dynamics
D	36	2.06	Cell cycle control, mitosis and meiosis
Y	1	0.06	Nuclear structure
V	86	4.93	Defense mechanisms
T	51	2.92	Signal transduction mechanisms
M	81	4.64	Cell wall/membrane biogenesis
N	14	0.8	Cell motility
Z	2	0.11	Cytoskeleton
W	0	0	Extracellular structures
U	36	2.06	Intracellular trafficking and secretion
O	68	3.9	Posttranslational modification, protein turnover, chaperones
C	98	5.62	Energy production and conversion
G	72	4.13	Carbohydrate transport and metabolism
E	111	6.36	Amino acid transport and metabolism
F	54	3.1	Nucleotide transport and metabolism
H	73	4.19	Coenzyme transport and metabolism
I	30	1.72	Lipid transport and metabolism
P	104	5.96	Inorganic ion transport and metabolism
Q	11	0.63	Secondary metabolites biosynthesis, transport and catabolism
R	204	11.7	General function prediction only
S	191	10.95	Function unknown
-	26	1.49	Not in COGs

^a^ The total is based on the total number of protein coding genes in the annotated genome.

## Insights into the genome sequence

There is a lack of closely related genomes because *Fenollaria* gen. nov. is a new genus. However, we made some comparisons against *Peptoniphilus sp.* oral taxon 386 str. F0131 (accession number NZ_GL349422), which is relatively close to *Fenollaria* based on 16S rRNA and for which the completed genome is available in public databases.

The draft genome sequence of *F. massiliensis* has a slightly bigger size compared to the *Peptoniphilus sp.*( 1.71 Mbp and 1.47 Mbp, respectively). The G+C content is slightly higher than *Peptoniphilus* sp. (34 and 31%, respectively). *Fenollaria massiliensis* gen. nov. encodes more genes (1,697 genes against 1,463 genes), however the number of genes per Mb is similar (1,007 – 1,004).

Table 6 presents the difference of gene number (in percentage) for each COG categories between *Peptoniphilus* sp. oral taxon 386 str. F0131 and *Fenollaria massiliensis* sp. nov.

**Table 6:** Percentage of genes associated with the 25 general COG functional categories for *Fenollaria massiliensis* and *Peptoniphilus sp.* oral taxon 386 str. F0131.

**Code**	**COG description**	***F. massiliensis*** **% of total**	***Peptoniphilus sp.*** **% of total**	**Difference (in %)**
J	Translation	9.86	10.01	1.5
A	RNA processing and modification	0.23	0.71	208.7
K	Transcription	6.25	6.52	4.3
L	Replication, recombination and repair	7.57	6.85	-9.5
B	Chromatin structure and dynamics	0.23	0.39	69.6
D	Cell cycle control, mitosis and meiosis	2.06	2.0	-2.9
Y	Nuclear structure	0.06	0	-100
V	Defense mechanisms	4.93	2.84	-42.4
T	Signal transduction mechanisms	2.92	2.97	1.7
M	Cell wall/membrane biogenesis	4.64	4.2	-9.5
N	Cell motility	0.8	1.1	37.5
Z	Cytoskeleton	0.11	0.19	72.7
W	Extracellular structures	0	0	0
U	Intracellular trafficking and secretion	2.06	2.84	37.9
O	Posttranslational modification, protein turnover, chaperones	3.9	4.26	9.2
C	Energy production and conversion	5.62	5.62	0
G	Carbohydrate transport and metabolism	4.13	2.65	-35.8
E	Amino acid transport and metabolism	6.36	7.56	18.9
F	Nucleotide transport and metabolism	3.1	3.94	27.1
H	Coenzyme transport and metabolism	4.19	2.78	-33.7
I	Lipid transport and metabolism	1.72	2.97	72.7
P	Inorganic ion transport and metabolism	5.96	4.78	-19.8
Q	Secondary metabolites biosynthesis, transport and catabolism	0.63	1.36	115.9
R	General function prediction only	11.7	11.56	-1.2
S	Function unknown	10.95	11.89	8.6
-	Not in COGs	1.49	1.29	8.6

Some COGs contain significantly more genes as “RNA processing and modification” (+208,7%) or “Secondary metabolites biosynthesis, transport and catabolism” (+115,9%), whereas others contain less genes as “Nuclear structure” (-100%) or “Defense mechanisms”(-42,4%).

## Conclusion

On the basis of phenotypic, phylogenetic and genomic analyses, we formally propose the creation of *Fenollaria massiliensis* gen. nov., sp. nov. that contains the strain 9401234^T^. This bacterium was found in Marseille, France.

### Description of *Fenollaria* gen. nov.

*Fenollaria* (Fe.nol.la′ria. N.L. gen. n. *Fenollaria* of F. Fenollar, expert microbiologist in Whipple’s disease and osteo-articular infections)

Gram negative rods. Obligate anaerobic. Non motile, non spore forming. Positive for indole. Negative for catalase and oxidase. Weakly positive gelatinase. Positive for leucine arylamidase, valine arylamidase, arginine arylamidase and for Naphtol phosphatase. Weakly positive for pyrrolidonyl arylamidase, tyrosine arylamidase, glycine arylamidase, histidine arylamidase and serine arylamidase. Habitat: human. Type species: *Fenollaria massiliensis*

### Description of *Fenollaria massiliensis* gen. nov. sp.nov.

*Fenollaria massiliensis* (ma.si.li.en′.sis. L. fem. adj. *massiliensis*, of *Massilia*, the Latin name of Marseille where was isolated *F. massiliensis*).

Gram negative, catalase negative, oxidase negative and obligate anaerobic. Cells are non-spore forming, non motile rods, with a mean length of 1,555 µm, and a mean width of 772 µm. Colonies are punctiform, very small, grey, smooth, and round on blood-enriched Columbia agar under anaerobic conditions. Optimal growth under anaerobic conditions, at 37°C (range from 32°C to 37°C). Cells are positive for leucine arylamidase, valine arylamidase, arginine arylamidase and for Naphtol phosphatase. Cells are weakly positive for pyrrolidonyl arylamidase, tyrosine arylamidase, glycine arylamidase, histidine arylamidase and serine arylamidase. Susceptible to penicillin G, amoxicillin, cefotetan, imipenem, metronidazole and vancomycin. The potential pathogenicity of the type strain 9401234^T^ is unknown.

The type strain is 9401234^T^ (= CSUR P127 = DSM 26367); it was isolated from an osteoarticular sample of a patient in Marseille (France). The G+C content of the genome is 34.46 mol%. A partial 16S rRNA gene sequence was deposited in GenBank with the accession number HM587321. The whole genome shotgun sequence of *F. massiliensis* strain 9401234^T^ (= CSUR P127 = DSM 26367) has been deposited in GenBank under accession numbers CALI02000001-CALI02000010.

## References

[1] La Scola B Fournier PE Raoult D Burden of emerging anaerobes in the MALDI-TOF and 16S rRNA gene sequencing era. Anaerobe 2011; 17:106-112 10.1016/j.anaerobe.2011.05.010 21672636

[2] Rossello-Mora R. DNA-DNA Reassociation Methods Applied to Microbial Taxonomy and Their Critical Evaluation. *In*: Stackebrandt E (ed), Molecular Identification, Systematics, and population Structure of Prokaryotes. Springer, Berlin, 2006, p. 23-50.

[3] Stackebrandt E Ebers J Taxonomic parameters revisited: tarnished gold standards. Microbiol Today 2006; 33:152-155

[4] Welker M Moore ER Applications of whole-cell matrix-assisted laser-desorption/ionization time-of-flight mass spectrometry in systematic microbiology. Syst Appl Microbiol 2011; 34:2-11 10.1016/j.syapm.2010.11.013 21288677

[5] Tindall BJ Rosselló-Móra R Busse HJ Ludwig W Kämpfer P Notes on the characterization of prokaryote strains for taxonomic purposes. Int J Syst Evol Microbiol 2010; 60:249-266 10.1099/ijs.0.016949-0 19700448

[6] Kokcha S Michra AK Lagier JC Million M Leroy Q Raoult D Fournier PE Non-contiguous-finished genome sequence and description of *Bacillus timonensis* sp. nov. Stand Genomic Sci 2012; 6:346-355 10.4056/sigs.2776064 23408487 PMC3558959

[7] Lagier JC El Karkouri K Nguyen TT Armougom F Raoult D Fournier PE Non-contiguous-finished genome sequence and description of *Anaerococcus senegalensis* sp. nov. Stand Genomic Sci 2012; 6:116-125 10.4056/sigs.2415480 22675604 PMC3359877

[8] Mishra AK Gimenez G Lagier JC Robert C Raoult D Fournier PE Non-contiguous-finished genome sequence and description of *Alistipes senegalensis* sp. nov. Stand Genomic Sci 2012; 6:304-314 10.4056/sigs.2625821 PMC3558963 23407265

[9] Lagier JC Armougom F Mishra AK Nguyen TT Raoult D Fournier PE Non-contiguous-finished genome sequence and description of *Alistipes timonensis* sp. nov. Stand Genomic Sci 2012; 6:315-324 10.4056/sigs.2685971 23408657 PMC3558960

[10] Mishra AK Lagier JC Robert C Raoult D Fournier PE Non-contiguous-finished genome sequence and description of *Clostridium senegalense* sp. nov. Stand Genomic Sci 2012; 6:386-395 23408737 10.4056/sigs.2766062 PMC3558962

[11] Michra AK Lagier JC Robert C Raoult D Fournier PE Non-contiguous-finished genome sequence and description of *Peptinophilus timonensis* sp. nov. Stand Genomic Sci 2012; (In press). 10.4056/sigs.2956294 PMC3570796 23449949

[12] Michra AK Lagier JC Robert C Raoult D Fournier PE Non-contiguous-finished genome sequence and description of *Peptinophilus senegalensis* sp. nov. Stand Genomic Sci 2012; (In press). 10.4056/sigs.3056450 PMC3577113 23459006

[13] Ludwig W, Schleifer KH, Whitman WB. Revised road map to the phylum *Firmicutes. In*: Bergey's Manual of Systematic Bacteriology, 2nd ed., vol. 3 (The Firmicutes) (P. De Vos, G. Garrity, D. Jones, N.R. Krieg, W. Ludwig, F.A. Rainey, K.-H. Schleifer, and W.B. Whitman, eds.), Springer-Verlag, New York. (2009) pp. 1-13.

[14] Mechichi T Labat M Garcia JL Thomas P Patel BK *Sporobacterium olearium* gen. nov., sp. nov., a new methanethiol-producing bacterium that degrades aromatic compounds, isolated from an olive mill wastewater treatment digester. Int J Syst Bacteriol 1999; 49:1741-1748 10.1099/00207713-49-4-1741 10555356

[15] Collins MD Shah HN Reclassification of *Bacteroides praeacutus* Tissier (Holdeman and Moore) in a new genus, *Tissierella*, as *Tissierella praeacuta* comb. nov. Int J Syst Bacteriol 1986; 36:461-463 10.1099/00207713-36-3-461

[16] Field D Garrity G Gray T Morrison N Selengut J Sterk P Tatusova T Thomson N Allen MJ Angiuoli SV The minimum information about a genome sequence (MIGS) specification. Nat Biotechnol 2008; 26:541-547 10.1038/nbt1360 18464787 PMC2409278

[17] Woese CR Kandler O Wheelis ML Towards a natural system of organisms: proposal for the domains *Archaea, Bacteria*, and *Eucarya.* Proc Natl Acad Sci USA 1990; 87:4576-4579 10.1073/pnas.87.12.4576 2112744 PMC54159

[18] Gibbons NE Murray RGE Proposals Concerning the Higher Taxa of Bacteria. Int J Syst Bacteriol 1978; 28:1-6 10.1099/00207713-28-1-1

[19] Garrity GM, Holt JG. The Road Map to the Manual. In: Garrity GM, Boone DR, Castenholz RW (eds), Bergey's Manual of Systematic Bacteriology, Second Edition, Volume 1, Springer, New York, 2001, p. 119-169.

[20] Murray RGE. The Higher Taxa, or, a Place for Everything...? In: Holt JG (ed), Bergey's Manual of Systematic Bacteriology, First Edition, Volume 1, The Williams and Wilkins Co., Baltimore, 1984, p. 31-34.

[21] List of new names and new combinations previously effectively, but not validly, published. List no. 132. Int J Syst Evol Microbiol 2010; 60:469-472 10.1099/ijs.0.022855-0 20458120

[22] Rainey FA. Class II. *Clostridia* class nov. In: De Vos P, Garrity G, Jones D, Krieg NR, Ludwig W, Rainey FA, Schleifer KH, Whitman WB (eds), Bergey's Manual of Systematic Bacteriology, Second Edition, Volume 3, Springer-Verlag, New York, 2009, p. 736.

[23] Skerman VBD Sneath PHA Approved list of bacterial names. Int J Syst Bact 1980; 30:225-420 10.1099/00207713-30-1-225

[24] Prevot AR. Dictionnaire des bactéries pathogens. *In*: Hauduroy P, Ehringer G, Guillot G, Magrou J, Prevot AR, Rosset, Urbain A (*eds*). Paris, Masson, 1953, p.1-692.

[25] Ashburner M Ball CA Blake JA Botstein D Butler H Cherry JM Davis AP Dolinski K Dwight SS Eppig JT Gene ontology: tool for the unification of biology. The Gene Ontology Consortium. Nat Genet 2000; 25:25-29 10.1038/75556 10802651 PMC3037419

[26] Farrow JAE Lawson PA Hippe H Gauglitz U Collins MD Phylogenetic evidence that the Gram-negative nonsporulating bacterium *Tissierella* (*Bacteroides*) *preacuta* is a member of the *Clostridium* subphylum of the Gram-positive bacteria, and description of *Tisiierella creatinini* sp. nov. Int J Syst Bacteriol 1995; 45:436-440 10.1099/00207713-45-3-436 8590669

[27] Tamura K Dudley J Nei M Kumar S MEGA4: Molecular Evolutionary Genetics Analysis (MEGA) software version 4.0. Mol Biol Evol 2007; 24:1596-1599 10.1093/molbev/msm092 17488738

[28] Murdoch DA Gram-positive anaerobic cocci. Clin Microbiol Rev 1998; 11:81-120 9457430 10.1128/cmr.11.1.81 PMC121377

[29] Seng P Drancourt M Gouriet F La Scola B Fournier PE Rolain JM Raoult D Ongoing revolution in bacteriology: routine identification of bacteria by matrix-assisted laser desorption ionization time-of-flight mass spectrometry. Clin Infect Dis 2009; 49:543-551 10.1086/600885 19583519

[30] Boetzer M Henkel CV Jansen HJ Butler D Pirovano W Scaffolding pre-assembled contigs using SSPACE. Bioinformatics 2011; 27:578-579 10.1093/bioinformatics/btq683 21149342

[31] Boetzer M Pirovano W Toward almost closed genomes with GapFiller. Genome Biol 2012; 13:R56 10.1186/gb-2012-13-6-r56 22731987 PMC3446322

[32] Lagesen K Hallin P Rodland EA Staerfeldt HH Rognes T Ussery DW RNAmmer: consistent and rapid annotation of ribosomal RNA genes. Nucleic Acids Res 2007; 35:3100-3108 10.1093/nar/gkm160 17452365 PMC1888812

[33] Laslett D Canback B ARAGORN, a program to detect tRNA genes and tmRNA genes in nucleotide sequences. Nucleic Acids Res 2004; 32:11-16 10.1093/nar/gkh152 14704338 PMC373265

[34] Griffiths-Jones S Bateman A Marshall M Khanna A Eddy SR Rfam: an RNA family database. Nucleic Acids Res 2003; 31:439-441 10.1093/nar/gkg006 12520045 PMC165453

[35] Bendtsen JD Nielsen H von Heijne G Brunak S Improved prediction of signal peptides: SignalP 3.0. J Mol Biol 2004; 340:783-795 10.1016/j.jmb.2004.05.028 15223320

[36] Hyatt D Chen GL Locascio PF Land ML Larimer FW Hauser LJ Prodigal: prokaryotic gene recognition and translation initiation site identification. BMC Bioinformatics 2010; 11:119 10.1186/1471-2105-11-119 20211023 PMC2848648

[37] Altschul SF Gish W Miller W Myers EW Lipman DJ Basic local alignment search tool. J Mol Biol 1990; 215:403-410 2231712 10.1016/S0022-2836(05)80360-2

